# Influence of the Apelinergic System on Conduction Disorders in Patients after Myocardial Infarction

**DOI:** 10.3390/jcm12247603

**Published:** 2023-12-10

**Authors:** Rafał Wyderka, Dorota Diakowska, Maria Łoboz-Rudnicka, Jakub Mercik, Michał Borger, Łukasz Osuch, Barbara Brzezińska, Anna Leśków, Małgorzata Krzystek-Korpacka, Joanna Jaroch

**Affiliations:** 1 Department of Cardiology, Tadeusz Marciniak Lower Silesia Specialist Hospital-Emergency Medicine Center, Fieldorf 2, 54-049 Wroclaw, Polandjakub.mercik@umw.edu.pl (J.M.); michal.borger@umw.edu.pl (M.B.);; 2 Faculty of Medicine, Wrocław University of Science and Technology, 50-370 Wroclaw, Poland; 3 Department of Basic Sciences, Faculty of Health Science, Wroclaw Medical University, Bartla 5, 51-618 Wroclaw, Poland; 4 Department of Internal Nursing, Faculty of Health Science, Wroclaw Medical University, Bartla 5, 51-618 Wroclaw, Poland; 5 Department of Medical Biochemistry, Wroclaw Medical University, Chalubinskiego 10, 50-368 Wroclaw, Poland

**Keywords:** myocardial infarction, coronary artery disease, apelinergic system, atrioventricular conduction disorders, Q/QRS ratio, elabela, apelin-13

## Abstract

Background: There is a growing body of evidence for an important role of the apelinergic system in the modulation of cardiovascular homeostasis. The aim of our study was to (1) examine the relationship between apelin serum concentration at index myocardial infarction (MI) and atrioventricular conduction disorders (AVCDs) at 12-month follow-up, and (2) investigate the association between initial apelin concentration and the novel marker of post-MI scar (Q/QRS ratio) at follow-up. Methods: In 84 patients with MI with complete revascularization, apelin peptide serum concentrations for apelin-13, apelin-17, elabela (ELA) and apelin receptor (APJ) were measured on day one of hospitalization; at 12-month follow-up, 54 of them underwent thorough examination that included 12-lead electrocardiography (ECG), Holter ECG monitoring and echocardiography. Results: The mean age was 58.9 years. At 12-month follow-up, AVCDs were diagnosed in 21.4% of subjects, with AV first-degree block in 16.7% and sinoatrial arrest in 3.7%. ELA serum concentration at index MI correlated positively with the occurrence of AVCD (*p* = 0.003) and heart rate (*p* = 0.005) at 12-month follow-up. The apelin-13 serum concentration at index MI correlated negatively with the Q/QRS ratio. Conclusions: The apelin peptide concentration during an acute phase of MI impacts the development of AVCD and the value of Q/QRS ratio in MI survivors.

## 1. Introduction

Coronary artery disease—a leading cause of global morbidity and mortality—results from atherosclerosis, which is a multifactorial, inflammatory process affecting the arterial wall. There is a growing body of evidence for an important role of the apelinergic system in the modulation of atherosclerotic process. The apelinergic axis consists of the peptide ligands apelin and elabela (ELA) and an apelin receptor (APJ). APJ belongs to the G-protein-coupled receptor family and resembles the type 1 angiotensin II receptor in terms of its structure, but is not activated by angiotensin II [1]. It is located in cardiomyocytes and endothelial and smooth muscle cells and is activated by various apelin isoforms and peptides of the ELA family [2]. Apelin peptides have been shown to exert various favorable effects on the cardiovascular system—they promote angiogenesis and vasodilatation, lower blood pressure, reduce fibrosis and cardiac hypertrophy and increase cardiac contractility and left ventricular stroke volume [3]. They also play a critical role in modulating response to ischemia as the activation of APJ during the acute phase of myocardial infarction (MI) promotes pro-survival cellular pathways and ischemia-induced angiogenesis and reduces expression of pro-inflammatory cytokines. The impairment of the function of the apelinergic axis causes the opposite and leads to unfavourable post-MI remodeling, which was shown in rodent models [4]. Most of the research on the relationship between the apelinergic system and cardiovascular physiology has until now been performed on animal models. Therefore, there is a strong need for studies in humans, examining the role of apelins in various cardiovascular diseases, including MI, as they could provide important information with clinical implications. Little is known about the relationship between the activation of the apelin axis in the acute phase of MI and the post-MI outcomes in humans. In our study, we aimed at investigating the correlation between the initial apelin peptide levels at index MI and atrioventricular conduction disorders (AVCDs) at 1-year follow-up. Furthermore, we presented a Q/QRS ratio—a novel, electrocardiographic marker of the post-MI scar size—and investigated the relationship between the initial apelin peptide concentration and the Q/QRS ratio at 1-year follow-up.

## 2. Materials and Methods

### 2.1. Study Population

This was a single-center, prospective study. A total of 84 patients, consecutively admitted with MI to the Tadeusz Marciniak Lower Silesia Specialist Hospital—Emergency Medicine Center, Department of Cardiology (Wroclaw, Poland), were enrolled into the study.

The exclusion criteria were as follows:

1. The history of previous MI or percutaneous coronary intervention;
2. MI treated conservatively or type II MI;
3. Chronic total occlusion (CTO) in coronary angiography;
4. Moderate to severe valvular disease;
5. The history of chronic kidney or liver disease (stage 4 and 5 of chronic kidney disease according to Kidney Disease Improving Global Outcomes criteria; diagnosed hepatic cirrhosis);
6. Advanced atrioventricular conduction disturbances.

All patients enrolled into the study underwent successful percutaneous coronary intervention.

At 12-month follow-up physical examination, 12-lead electrocardiography (ECG), 12-channel Holter ECG and echocardiography were performed. Due to the incomplete data, 30 patients were excluded from further analysis.

### 2.2. Biochemical Determinations

Venous blood samples (2 mL) were collected on the first day of hospitalization using a vacuum system. Blood samples were stored in tubes with EDTA and coagulated for 30 min at room temperature, then centrifuged at 3000× *g* (centrifuge MPW 260R, MPW Med. Instruments, Warsaw, Poland) for 15 min at room temperature. The obtained sera were stored at −20 °C.

ELA, apelin-13 (AP-13), apelin-17 (AP-17) and APJ receptor concentrations were measured using ELISA sets (MyBioSource Inc., San Diego, CA, USA) in accordance with the manufacturer’s instructions. The sensitivity of the ELA test was 10.0 pg/mL. The sensitivity of the APJ receptor was 7.4 pg/mL, while the intra- and inter-assay CV values were <8.0% and <10.0%, respectively. The sensitivity of the AP-13 assay was less than 33.0 pg/mL, and the intra- and inter-assay CVs were <4.0% and <6.5%, respectively. The minimum detectable dose of AP-17 was less than 72.0 pg/mL, and intra- and inter-assay CVs were <10.0% and <12.0%, respectively.

Hematological variables data, glucose, total cholesterol (TCh), HDL and LDL cholesterol, triglycerides (TG), C-reactive protein (CRP), high-sensitivity troponin T (Hs-Troponin T), myocardial creatinine kinase (CK) (band—MB) and N-terminal pro-B-type natriuretic peptide (NT-proBNP) were measured in blood samples collected on hospital admission.

### 2.3. Electrocardiographic Parameters

At the follow-up performed 12 months after the MI hospitalization physical examination, 12-lead ECG, 12-channel Holter ECG and echocardiography were performed. The artifacts of the Holter ECG records were removed manually. Analyzed parameters were calculated using BTL CardioPoint^®^ Holter H600 software, v. 2.30.33006.0. Sinoatrial arrest was defined as a pause in sinus activity > 2 s. Atrioventricular (AV) block was defined according to general electrocardiographic criteria: (1) first-degree AV block as PR interval on ECG > 200 ms; (2) second-degree AV block as a drop in QRS complex (Mobitz I—preceded by a prolongation of PR interval; Mobitz II—without prior prolongation of PR interval); and (3) third-degree AV block as a complete disruption of AV conduction with ventricular electrical activity not related to and slower than the atrial electrical activity. Q/QRS ratio was measured manually and the greatest available ratio was taken into consideration.

### 2.4. Statistical Analysis

Descriptive data were presented as numbers of observations and percentages; mean, median, standard deviation (±SD), and quartile (Q1–Q3) values were calculated. The normality of the data distribution was examined using the Shapiro–Wilk test.

In the study, we built a multiple regression model with ECG parameters as dependent variables and demographic (age, sex), clinical (BMI, hypertension, diabetes type 2, Syntax score, atrial fibrillation, type of myocardial infarction), biochemical (troponin, NT-proBNP, CRP serum levels) and echocardiographic (left ventricular ejection fraction, left ventricular mass index) baseline parameters as predictors of ECG factors.

Initially, correlation analyses were performed between the variables (parametric Pearson test or non-parametric Spearman test, respectively) in order to determine the predictors most closely associated with the ECG parameters and exclude predictor collinearity. Subsequently, linear regression analysis and multiple regression models were constructed for quantitative dependent ECG variables. In linear regression analysis, an exclusion criterion was *p* > 0.100. Comparative analyses were performed using the Student *t*-test and Mann–Whitney test (depending on the type of variable distribution), which showed no significant differences in the concentrations of ELA, AP-13, AP-17 and APR between the dichotomous ECG variables.

Two-tailed *p*-values < 0.05 were assumed as statistically significant. Statistical analyses were conducted using Statistica 13.3 software (Tibco Inc., Palo Alto, CA, USA).

## 3. Results

Detailed baseline characteristics of the initial study group (n = 84) and the study group that completed the 12-month follow-up (n = 54) are presented in Table S1. The mean age of the population that completed the 12-month follow-up was 58.9 years. The majority of subjects were males (77.8%), and the most common type of myocardial infarction was STEMI (70.4%), mainly in the anterior territory (61% of STEMI pts). Angiography revealed one-vessel coronary disease in 50%, two-vessel in 38.9% and three-vessel in 11.1% of patients, with a mean Syntax score of 16.9 points.

At 12-month follow-up, almost all of the patients had sinus rhythm (98.1%) with a mean heart rate of 67.7 bpm. Conduction disorders were diagnosed in 21.4% of subjects: first-degree AV block in 16.7% and sinoatrial arrest in 3.7% (Table S1). Electrocardiographic and Holter ECG characteristics in patients after MI are shown in Table 1.

Final models of multiple linear regressions with selected predictors of ECG parameters are presented in Table 2. The mean heart rate at 12-month follow-up was inversely correlated with the patient’s age (*p* = 0.002) and positively correlated with ELA serum concentration at index MI (*p* = 0.005). The occurrence of atrioventricular conduction disorders (first-degree AV block and sinoatrial arrest) at follow-up correlated positively with ELA serum concentration at index MI (*p* = 0.003) and diabetes (*p* = 0.028).

The Q/QRS complex at follow-up correlated negatively with the apelin-13 serum concentration at index MI (*p* = 0.023) (Table 3).

## 4. Discussion

There are few data in the literature concerned with the influence of the apelin axis on conduction disorders and—to the best of our knowledge—this is the first study that investigated the relationship between apelin peptide levels during the acute phase of MI and the development of atrioventricular conduction disorders at follow-up. In our study, we demonstrated a significant positive relationship between ELA serum concentration at index MI and the occurrence of atrioventricular conduction disorders at 12-month follow-up. The possible explanation for this phenomenon might be that enhanced upregulation of the apelin axis during acute phase of MI reflects more extensive cardiac damage with greater compensatory response and hence predicts unfavorable outcomes at follow-up, including atrioventricular conduction disorders. Donmez et al. showed that in patients with STEMI, ELA serum level is positively associated with troponin and NT-proBNP concentration and inversely with left ventricular ejection fraction, which may suggest its increased concentration in cases of more severe ischemia [5]. The activation of the apelin peptides on the first day of MI has also been shown in previous reports [6,7]. The mechanism of upregulation of the apelinergic system was also postulated in different clinical scenarios—in a report by Acele et al., increased ELA serum levels were found in subjects with complete atrioventricular block in comparison to healthy controls [8]. The authors speculated that increased ELA levels reflected the upregulation of the apelin axis in response to bradycardia, which was an important part of the mechanism involved in maintaining the cardiac output.

The next finding of our study is the positive relationship between ELA serum concentration at index MI and heart rate at 1-year follow-up. The evidence for some direct relationship between apelinergic system components and heart rate was provided by experimental studies performed in animals, which demonstrated that administration (intravenous, intracerebroventricular, intraperitoneal, etc.) of apelins causes an immediate increase in heart rate [9,10,11]. Apelins seem to exert positive chronotropic effects through the autonomic nervous system, as their immunoreactivity has been detected in the hypothalamus and brain stem [9,12]. However, the explanation for the relationship between the ELA concentration at index MI and heart rate assessed at 1 year could be more complex and requires further research. Heart rate is an important prognostic factor in patients after MI. Furthermore, it has been shown that heart rate at 1-year follow-up is an even more powerful predictor of mortality than HR at index MI [13].

Another important aspect of our work is the relationship between apelin-13 level at index MI and the Q/QRS ratio at 1-year follow-up. Although it is cardiac magnetic resonance that is a reference method for assessing post-infarct scars, the Q/QRS ratio is a simple, novel parameter that may serve as an electrocardiographic indicator of the size of the post-MI scar—the more extensive the scar, the greater the Q/QRS ratio. Due to the high availability of the ECG test and the simplicity of the measurement of the Q/QRS ratio, it may become an important prognostic factor in patients after MI. To the best of our knowledge, this is the first paper presenting the Q/QRS ratio.

Apelins exert powerful cardioprotective and proangiogenic effects and the loss of their function in the scenario of MI is associated with increased infarct size and adverse cardiac remodeling, which was elegantly shown in a murine model by Wang et al. [4]. The results of our study are consistent with this previous observation, as we demonstrated that a decreased concentration of apelin-13 serum level at index MI is related to an increased Q/QRS ratio at 1-year follow-up. In addition, the increased Q/QRS ratio positively correlated with the presence of atrioventricular conduction disorders (in our study, first-degree AV block and sinoatrial arrest), which can be explained by a greater myocardial damage and thus a greater risk of damage to the conduction system.

This strong correlation between the Q/QRS ratio and atrioventricular conduction disorders may be an important factor promoting the careful observation of and search for conduction disorders in patients after a recent MI, who initially did not have the above-mentioned disorders but who presented with an increased Q/QRS ratio at follow-up.

### Strengths and Limitations

The major strength of the study was the measurement of serum concentrations of the novel apelin axis peptides in humans in the scenario of myocardial infarction. The limitations are as follows: It was a single-center study. The study group was relatively small and consisted only of a Caucasian population.

## 5. Conclusions

Initial ELA concentration at index MI predicts atrioventricular conduction disorders in MI survivors at follow-up.

Decreased apelin serum concentration during the early phase of MI is related to increased Q/QRS ratio—a novel marker of the size of the post-MI scar.

## Figures and Tables

**Table 1:** Electrocardiographic parameters of patients 12 months after MI. Descriptive data are presented as number of observations (%), mean ± SD, median [Q1; Q3].

	Study Group (n = 54)	
Heart rhythms:	sinus	53 (98.1)
	atrial fibrillation	1 (1.9)
Heart rate (mean, beats per minute)		67.7 ± 7.5
Electric axis:	Indirect	36 (66.7)
	Left axis	13 (24.1)
	Right axis	4 (7.4)
	Undefined	1 (1.9)
PQ min. (ms)		121.1 ± 17.5
PQ max (ms)		227.1 ± 41.7
PQ average (ms)		179.8 ± 27.8
Significant pauses:	Yes	2 (3.7)
	No	52 (96.3)
Atrioventricular conduction disturbances:	No	43 (79.6)
	Sinoatrial arrest	2 (3.7)
	AV block I degree	9 (16.7)
	AV block II degree	0 (0.0)
QRS time (ms)		103.5 ± 16.2
Q/QRS proportion (%)		33.3 [0.0; 100.0]

**Table 2:** Final models of multiple linear regressions with selected predictors of ECG parameters. Selection was performed according to significant results of apelinergic system components in linear regression analyses (*p* ≤ 0.100).

**Mean heart rate**						
Model	B	SE	β (beta)	*t*-value	*p*-value	R^2^
constant	67.65	9.40	-	7.19	<0.0001	0.32
Male sex	3.75	2.24	0.21	1.67	0.101	
Age (years)	−0.28	0.09	−0.39	−3.17	0.002 *	
Diabetes mellitus type II	3.57	2.47	0.17	1.44	0.155	
ELA (pg/mL)	0.01	0.00	0.35	2.93	0.005 *	
**Atrioventricular conduction disturbances**						
Model	B	SE	β (beta)	*t*-value	*p*-value	R^2^
Constant	−1.99	0.73	-	−2.72	0.008	0.23
Diabetes mellitus type 2	0.61	0.27	0.29	2.26	0.028 *	
ELA (pg/mL)	0.001	0.0005	0.41	3.15	0.003 *	
CRP (mg/L)	−0.003	0.002	−0.17	−1.26	0.212	

*: statistically significant; ELA: elabela peptide; CRP: C-reactive protein.

**Table 3:** Final models of multiple linear regressions with selected predictors of ECG parameters. Selection was performed according to significant results of apelinergic system components in linear regression analyses (*p* ≤ 0.100).

Q/QRS Proportion (%)						
Model	B	SE	β (beta)	*t*-value	*p*-value	R^2^
Constant	119.78	30.16	-	3.97	0.0002	0.11
Male sex	13.79	12.32	0.13	0.96	0.340	
Diabetes mellitus type 2	17.16	17.60	0.14	0.97	0.334	
AP-13 (pg/mL)	−0.89	0.38	−0.33	−2.34	0.023 *	

*: statistically significant. AP-13: apelin-13.

## Data Availability

The data are available from the corresponding author and may be shared if necessary.

## Supplementary Materials

The following supporting information can be downloaded at https://www.mdpi.com/article/10.3390/jcm12247603/s1, Table S1: Basic demographic, clinical and laboratory data.
