# Supplementary material for: Influence of the Apelinergic System on Conduction Disorders in Patients after Myocardial Infarction
Source: J Clin Med. 2023 Dec 10;12(24):7603. doi: 10.3390/jcm12247603 (PMC10744328; doi:10.3390/jcm12247603)
Supplement: Supplementary file 1 [file jcm-12-07603-s001.zip › jcm-2711878-supplementary.pdf]

Table S1. Basic demographic, clinical and laboratory data.

		Study group with follow-up (n=54)	Initial study group (n=84)
Age (years)		58.9 ± 10.2	58.7 ± 10.1
Sex: male/female (number/%)		42 (77.8) / 12 (22.2)	64 (76.2)/ 20 (24.8)
BMI (kg/m^2)		28.9 ± 4.6	27.8 ± 4.5
CHA_2DS_2-VASc score		2.6 ± 1.2	3.0 ± 1.4
SYNTAX score		16.9 ± 8.2	18 ± 9.3
Coronary artery disease (CAD): (number/%)	one-vessel CAD<br>two-vessel CAD<br>three-vessel CAD	27 (50.0)<br>21 (38.9)<br>6 (11.1)	34 (40.5)<br>35 (41.7)<br>15 (17.8)
Hypertension: (number/%)	yes<br>no	36 (66.7)<br>18 (33.3)	58 (69.0)<br>26 (31.0)
Diabetes mellitus type 2: (number/%)	yes<br>no	8 (14.8)<br>46 (85.2)	16 (19.0)<br>68 (81.0)
Atrial fibrillation (AF): (number/%)	yes<br>no	3 (5.6)<br>51 (94.4)	5 (6.0)<br>79 (94.0)
LVEF (%)		45.04 ± 7.81	45.0 ± 8.12
LVEF ≤ 35% (number/%)	yes<br>no	7 (12.9)<br>47 (87.1)	13 (15.5)<br>71 (84.5)
Time of hospitalization (days)		6.02 ± 1.74	6.0 ± 2.3
Type of myocardial infarction:	NSTEMI<br>STEMI	16 (29.6)<br>38 (70.4)	26 (31.0)<br>58 (69.0)
Localization of MI based on the ECG:	Anterior wall (V1-V4)<br>Lateral wall (I, aVL, V5, V6)<br>Inferior wall (II, III, aVF)<br>No data	33 (61.1)<br>3 (5.6)<br>14 (25.9)<br>4 (7.4)	33 (39.3)<br>3 (3.6)<br>14 (16.7)<br>4 (4.8)<br>30 (35.8)

Laboratory parameters:		
CK-MB (IU/L)	82.5 [46.0; 164.0]	82.5 [44.5; 160.0}
Hs Troponin T (ng/L)	1864.0 [1052.0, 4792.0]	1881.0 [843.0; 4469.0]
CRP (mg/L)	2.7 [1.0; 5.7]	3.25 [1.05; 7.79]
NT-proBNP (pg/mL)	292.0 [70.9; 766.0]	361.0 [79.0; 975.2]
ELA (pg/mL)	1471.5 ± 199.2	1462.5 ± 229.8
AP-13 (pg/mL)	71.5 ± 16.5	67.2 ± 16.0
AP-17 (pg/mL)	624.9 ± 159.5	586.6 ± 168.7
APR (pg/mL)	1432.2 ± 203.3	1346.8 ± 205.0

BMI: body mass index; LVEF: left ventricular ejection fraction; CK-MB: creatine kinase-myocardial band; Hs Troponin T: high-sensitivity troponin T; CRP: C-reactive protein; NT-proBNP: N-terminal-proB-type natriuretic peptide; ELA: elabela peptide; AP-13: apelin-13; AP-17: apelin-17; APR: apelin receptor
